# A Broader Perspective on Anti-Ro Antibodies and Their Fetal Consequences—A Case Report and Literature Review

**DOI:** 10.3390/diagnostics10070478

**Published:** 2020-07-14

**Authors:** Mihaela Roxana Popescu, Andreea Dudu, Ciprian Jurcut, Anca Marina Ciobanu, Ana-Maria Zagrean, Anca Maria Panaitescu

**Affiliations:** 1 Cardiology Department, Elias University Hospital, “Carol Davila” University of Medicine and Pharmacy, 011461 Bucharest, Romania; 2 Internal Medicine Department, “Dr Carol Davila” Central Emergency University Military Hospital, 010825 Bucharest, Romania; andreear91@yahoo.com (A.D.); cjurcut@gmail.com (C.J.); 3 Department of Obstetrics and Gynecology, Filantropia Clinical Hospital, “Carol Davila” University of Medicine and Pharmacy, 011171 Bucharest, Romania; ciobanu.ancamarina@gmail.com (A.M.C.); panaitescu.anca@yahoo.com (A.M.P.); 4 Division of Physiology and Neuroscience, Department of Functional Sciences, “Carol Davila” University of Medicine and Pharmacy, 050474 Bucharest, Romania; ana-maria.zagrean@umfcd.ro

**Keywords:** anti Ro/SSA antibodies, fetal complete heart block, long-term consequences, apoptosis, autoimmune channelopathy

## Abstract

The presence of maternal Anti-Ro/Anti-La antibodies causes a passively acquired autoimmunity that may be associated with serious fetal complications. The classic example is the autoimmune-mediated congenital heart block (CHB) which is due in most cases to the transplacental passage of Anti-Ro/Anti-La antibodies. The exact mechanisms through which these pathologic events arise are linked to disturbances in calcium channels function, impairment of calcium homeostasis and ultimately apoptosis, inflammation and fibrosis. CHB still represents a challenging diagnosis and a source of debate regarding the best management. As the third-degree block is usually irreversible, the best strategy is risk awareness and prevention. Although CHB is a rare occurrence, it affects one in 20,000 live births, with a high overall mortality rate (up to 20%, with 70% of in utero deaths). There is also concern over the lifelong consequences, as most babies need a pacemaker. This review aims to offer, apart from the data needed for a better understanding of the issue at hand, a broader perspective of the specialists directly involved in managing this pathology: the rheumatologist, the maternal–fetal specialist and the cardiologist. To better illustrate the theoretical facts presented, we also include a representative clinical case.

## 1. Introduction

### 1.1. What Exactly Do the Anti-Ro/SSA and Anti-La/SSB Antigen–Antibody Systems Represent?

Anti-Ro/Sjögren’s-syndrome-related antigen A (SSA) autoantibodies are directed against autoantigens Ro52 and Ro60, represented by distinct cellular proteins of 52 and 60 kDa, respectively.

*The Ro60 antigen*, also known as TROVE2, was first described in 1988 by Deutscher et al. [1] and is located in the nucleus and nucleolus. Studies on mice have shown that the absence of a functional gene encoding Ro60 makes the animal susceptible to systemic autoimmune diseases, characterized by the development of anti-ribosome and anti-chromatin antibodies, accompanied by glomerulonephritis [2].

*The Ro52 autoantigen (TRIM21),* first mentioned in 1991 [3], is an interferon-inducible protein residing in the cytoplasm, known for its important role in the regulation of inflammation. For instance, Ro52 inhibits the inflammatory process by targeting interferon regulatory factors three and seven, as well as by downregulating proinflammatory nuclear factor kappa-B [4,5,6]. It has been shown that autoimmune diseases manifested by hypergammaglobulinemia and renal disease are present in mice lacking Ro52 [7]. In addition, the presence of autoantibodies to Ro52 was shown to accompany cardiac injury [8].

Anti-La/Sjögren’s-syndrome-related antigen B (SSB) autoantibodies target a 47-kDa protein which can be found between the nucleus and cytoplasm, but mostly in the nucleus [9]. The main role of La/SSB antigen is to regulate the stability of messenger (m)RNA [10].

### 1.2. Preferred Methods in the Detection of Anti-Ro and Anti-La Antibodies

Anti-Ro and anti-La antibodies are nowadays mainly detected using solid-phase assays, such as enzyme-linked immunosorbent assays (ELISA) or antigen-coated fluorescent microsphere and flow cytometry-based assays. Although these methods have many advantages such as a decreased cost, the ability to quantify the amount of antibody and increased sensitivity, we should keep in mind the fact that their specificity for the diagnosis of autoimmune diseases is quite low [11,12].

### 1.3. Autoimmune Disorders with Detectable Anti-Ro Antibodies

The presence of anti-Ro/SSA and anti-La/SSB antibodies was initially related to patients with Sjögren’s syndrome (SS) and systemic lupus erythematosus (SLE), but further studies have proven that anti-Ro antibodies can be detected in many other autoimmune diseases [12,13,14,15,16,17]. On the other hand, anti-La antibodies are still regarded as being specific in the diagnosis of SLE and SS.

As already discussed above, the “Ro” autoantigen consists of two proteins (weighing 60 kDa and 50 kDa) which can be detected using separate solid-phase immunoassays. Moreover, the presence of anti-Ro52 and anti-Ro60 antibodies in the serum of patients may have different clinical significance. For instance, a combination of these two can be frequently found in patients with SS. Furthermore, the specificity of anti-Ro antibodies for this disease, makes them a part of the ACR-EULAR Classification Criteria for primary SS [18].

Another important clinical scenario is represented by the situation in which asymptomatic patients carry different titers of anti-Ro antibodies [19,20,21]. Their presence can also precede the onset of an autoimmune disease. Clinical studies have shown that anti-Ro antibodies were detectable in 48% of the patients with known SLE, prior to the diagnosis. Furthermore, it is estimated that the average time interval between the detection of the antibodies and the diagnosis of SLE is around 3.6 years [22]. When it comes to SS, experts observed that it takes around four years from the moment when positive antibodies are first detected in asymptomatic patients and the time of a SS diagnosis [23].

### 1.4. Who Should Be Tested for the Presence of Anti-Ro Antibodies?

The patients with anti-Ro antibodies can be symptomatic or completely asymptomatic. There are some women at risk of having positive anti-Ro and/or anti-La antibodies especially when diseases like SLE, SS, rheumatoid arthritis or mixed connective tissue disease have already been diagnosed. Women with neonatal lupus and/or cardiac manifestations in a previous pregnancy are also at risk for subsequent complicated pregnancies. As a result, it is recommended that these categories of mothers should be tested before conception or as soon as possible during the pregnancy, even if the absence of these antibodies has been proved in the past. Even asymptomatic mothers with slow fetal heart rate and echocardiographic findings of heart block should also be tested for the presence of these antibodies, as it is not unusual for neonatal lupus to be the first clue toward the detection of anti-Ro/SSA and anti-La/SSB antibodies (see also the clinical case report and Table 1).

The major issue at hand is that the anti-Ro antibodies are detectable in up to 3% of the general population [20]. This translates into a significant number (approx. 700/year in the United States) of potential congenital heart block (CHB) cases. Still, a large proportion of the anti-Ro positive, asymptomatic women will get pregnant without ever being tested [25].

The purpose of screening for these antibodies is to avoid as many pregnancies in women that are unaware of their anti-Ro-positive status as possible and offer the best monitoring possible.

## 2. Pregnancy in Women with Anti-Ro/SSA Antibodies

The main concern regarding women with positive anti-Ro/SSA antibodies is the risk of giving birth to children with neonatal lupus, which leads not only to cutaneous or hematological manifestations (anemia, thrombocytopenia), but also to consequences such as CHB. The fetal and neonatal long-term outcomes were analyzed in a large observational study which included 325 children with second or third degree heart block of women with confirmed anti- Ro, La antibodies. The overall mortality rate was 17.5%. The risk of intrauterine death was 6% and the overall survival rate at 10 years was 86%. Seventy percent of children required pacing by 10 years of age [25]. Predictive factors of intrauterine death were hydrops and earlier gestational age at the time of cardiac manifestation onset, while predictive factors of neonatal death were hydrops and endocardial fibroelastosis [26]. However, in cases of isolated congenital heart block the rate of mortality reported in the literature varies between 9% and 25% for antenatal mortality rate and between 9% and 15% for neonatal mortality rate [27,28,29,30]. Fetal hydrops and early gestational age at diagnosis are consistent predictive factors of intrauterine death.

While pregnancies resulting in fetal complications from mothers with high levels of anti-Ro and anti-La are more common, there are only 14 reported cases of autoimmune mediated CHB from mothers with only anti-La antibodies [31]. This confirms the fact that they should not be used to predict autoimmune CHB. If there is a distinct role of these antibodies in the pathogenesis of CHB, it still needs to be elucidated.

### 2.1. Frequency of Gestational Complications

The risk of developing a *congenital fetal heart block* varies between 0.2–2% in nulliparous women with positive anti-Ro antibodies, and increases to 15–20% in pregnancies with a previously affected fetus or neonate [25,32]. Women who had two previously affected pregnancies have a risk of 50% of fetal CHB in subsequent pregnancies [31].

For anti-Ro antibody titers higher than 50 UI/mL, the risk of developing CHB is 5%, with high risk of developing a complete atrioventricular (AV) block in the fetus [33]. It is important to know that asymptomatic mothers with higher titers of anti-Ro antibodies (without symptoms or signs of SLE and/or SS) can give birth to children with neonatal lupus. However, studies show that about one-half of these women will later develop an autoimmune disease, especially SS [34].

Cutaneous manifestations of neonatal lupus seem to appear in 7–16% of the newborns with anti-Ro and anti-La positive mothers, and can be associated with the presence of anti-ribonucleoprotein (RNP) antibodies [35].

### 2.2. Molecular Mechanisms Leading to Fetal Complications

The precise molecular mechanism through which the anti-Ro/SSA and anti-La/SSB antibodies affect the fetal skin and heart is still an ongoing research topic. The presence of anti-Ro antibodies is necessary, but not sufficient to cause CHB. Current studies have shown that the development of neonatal lupus is influenced by the interaction between the transplacental passage of maternal antibodies, starting in the late first trimester or early second trimester and additive factors such as fetal genetic (specific human leukocyte antigen (HLA) alleles) and environmental factors. Such interactions could explain why these complications still remain rare, as the presence of even high titers of these antibodies alone is not enough to cause the disease (as previously mentioned, the maximum risk is 2% in nulliparous women) [36]. This theory is also supported by the discordance of heart block in identical twins, which suggests that in utero factors have also an important role to play in the pathogenesis of CHB [37].

Available immunohistochemistry data confirms that the permanent electrogenic disturbances are ultimately caused by fibrosis and calcification, but the ultimate pathway to fibrosis may be variable [38]. There are two theories regarding the molecular mechanism of the autoimmune induced CHB, the “apoptosis hypothesis” and the “Ca channel hypothesis” [39,40,41].

#### 2.2.1. The “Apoptosis Hypothesis”

**Figure 1:**
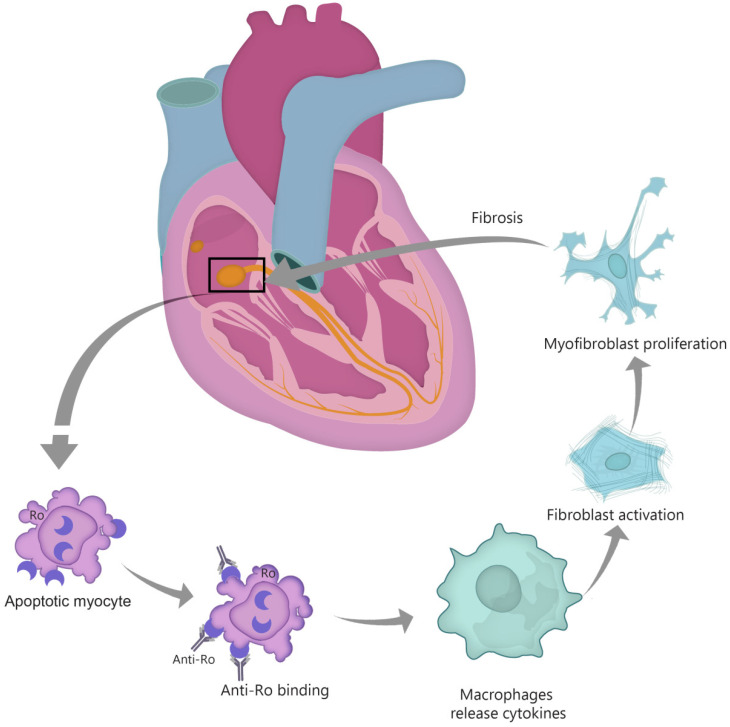
“Apoptosis hypothesis”. TGF-β—transforming growth factor β.

The first theory suggests that anti-Ro/SSA antibodies bind to surface antigens of cells undergoing apoptosis in the process of physiological remodeling (see Figure 1). Typically, Ro antigens are intracellular, but the process of apoptosis induces translocation of these antibodies to the surface of fetal cardiomyocytes. In normal conditions, the apoptotic cardiocytes are phagocytosed, but the immune complexes impair the clearance of these cells [42]. Apoptotic cell accumulation induces macrophage infiltration, activation and release of cytokines such as TNF-α and TGF-β [31,38,42,43,44]. TGF stimulates the differentiation of fibroblasts into myofibroblasts, leading to scar formation [38]. Thus, the fetal cardiac conduction tissue is exposed to an autoimmune reaction that causes inflammation and fibrosis, leading to irreversible CHB [39]. Interestingly, in children with autoimmune CHB, a certain polymorphism of the TGF gene was described, linked to increased fibrosis [45]. Moreover, data from in vitro studies show that anti-Ro–associated single-stranded RNA binds to macrophage Toll-like receptors (TLR) and thus triggers the aforementioned inflammatory and fibrosis cascade [46]. An important role seems to be played in this context by interferon (IFN), with highly expressed type I IFN response genes [46,47].

#### 2.2.2. The “Calcium Channel Hypothesis”

**Figure 2:**
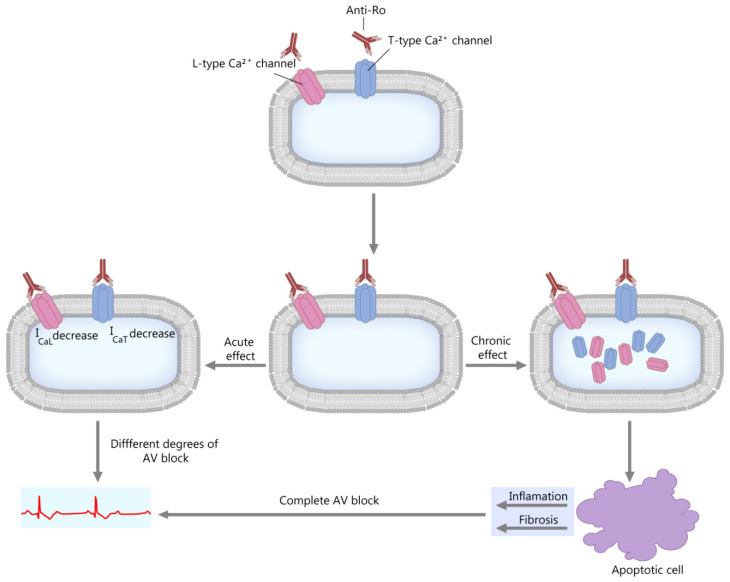
“Calcium hypothesis”. The short-term and long-term effects. AV block—atrioventricular block; I_CaL_; I_CaT_—calcium current through L-type and T-type Ca channels.

This theory is centered on the fact that the anti-Ro antibodies inhibit the calcium channels by direct interaction [39,48,49,50]. The anti-Ro/SSA antibodies can inhibit both the L-type and T-type calcium channels [39,51], which are essential to impulse propagation and conduction in the sinoatrial and AV nodes [43,51,52,53]. As a consequence, a short-term effect is a decrease in calcium currents, and a long-term effect is the internalization of the calcium channels (see Figure 2). These events lead to a disruption of the intracellular calcium handling, possibly contributing to the aforementioned apoptotic process and subsequent inflammation and fibrosis [39,50,54].

### 2.3. Is There a Relationship between the Titers of Anti-Ro/SSA and/or Anti-La/SSB Antibodies and the Incidence of Congenital Heart Block?

As previously discussed, experimental data show that the development of CHB is more frequent in newborns of women with high titers of anti-Ro/SSA and anti-La/SSB antibodies. In a prospective study of 186 antibody-exposed fetuses and newborns, Jaeggi E. et al. observed that the presence of maternal anti-Ro antibodies is responsible for the fetal tissue injury in a dose-dependent manner. The cutoff value was 50 U/mL, as measured by ELISA assay. As a result, many experts are recommending serial echocardiography only in women with high anti-Ro titers [33].

Another study conducted by Kan N. et al. including 232 pregnancies of anti-Ro antibodies positive mothers, proved that limiting serial fetal echocardiograms to women with high anti-Ro antibody levels is safe and cost-effective [55].

### 2.4. Clinical Manifestations of Neonatal Lupus

The two major clinical manifestations of neonatal lupus can be divided into cutaneous (typical rash) and cardiac findings.

#### 2.4.1. Cutaneous Manifestations

The rash is frequently observed within a few weeks after birth (sometimes up to four months), but it can also be noticed at birth, which proves that its photosensitivity is not a mandatory characteristic [56]. Neiman A.R. et al. concluded that the rash is usually diagnosed around six weeks and it is self-limiting. It almost always disappears by six to eight months, lasting for an average of 17 weeks [57].

Moreover, the exposure to ultraviolet light is thought to induce or exacerbate the rash. It usually manifests with annular lesions or arcuate macules that are mainly located on the scalp and in the periorbital area, but also on other parts of the body such as the palms, the soles and the diaper area [58].

Another rare cutaneous manifestation is telangiectasia on the face or genitals. It can be observed in about 10% of patients and can occur between 6 to 12 months of age, not only as the first manifestation of the disease, but also localized in skin areas that were previously affected by the typical rash [59].

It is noteworthy that patients with cutaneous neonatal lupus are at increased risk of developing autoimmune diseases throughout their lives and this is the reason why some authors are recommending a continuous follow-up, especially prior to adolescence [60].

#### 2.4.2. Cardiac Manifestations

The risk of developing first-, second- or third-degree AV block is one of the most threatening complications of the presence of anti-Ro/SSA antibodies. It commonly occurs between 18 to 24 weeks of gestation, which explains why mothers at risk should undergo more intensive fetal surveillance in this delicate period.

Isolated cases have been reported as early as 16 weeks, although another systematic review showed that 75% of cases were diagnosed during weeks 20 to 29 [31].

**Figure 3:**
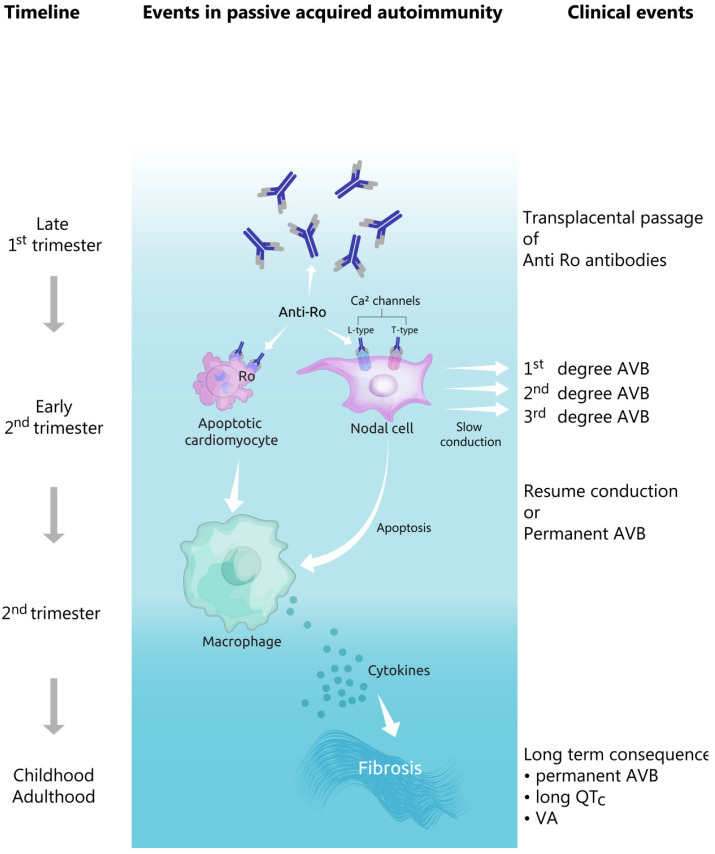
Evolution of pathophysiological and clinical events in the development of congenital heart block. AVB—atrioventricular block, VA—ventricular arrhythmias.

The first-degree AV block (defined by a PR interval which is longer than the upper limits of normal age) is considered to be a benign ECG finding, which appears in up to 6% of normal neonates and doesn’t lead to fetal bradycardia [61]. However, further fetal surveillance is recommended, as the first, as well as the second-degree AV blocks, may progress to more advanced blocks (see Figure 3). In addition, the autoimmune reaction affects the calcium channels present not only in the AV node, but also in the sinoatrial node. In this case, sinus node bradycardia may ensue.

The second-degree AV block may lead to fetal bradycardia, especially in advanced heart blocks in which two consecutive P waves fail to conduct to the ventricle, suggesting the presence of an important conduction block below the level of the AV node [62].

The third degree (or complete) AV block is the most serious and dramatic manifestation of neonatal lupus which leads to complete dissociation of the atrial and ventricular activity, as a result of the damage in the AV conduction pathways. In patients with third-degree AV block the atrial frequency is in the normal range, but the ventricular rate can vary between 50 and 80 beats/minute, as the rhythm is junctional or ventricular.

The resulting fetal bradycardia increases the risk of heart failure and sudden cardiac death. It is also responsible for the poor systemic perfusion, which can lead to hydrops fetalis, as a result of impaired ventricular filling and/or cardiac output [62,63]. Hydrops fetalis is defined as a collection of fluid in two body cavities (pleural, pericardial, ascites) or one body cavity plus anasarca [63,64].

The autoantibody-mediated heart blocks are usually associated with a structurally normal heart, although valvular lesions have been reported by some authors. For example, Cuneo BF et al. described three fetuses with ”unique” myocardial and conduction system diseases such as mitral and tricuspid valve chordal avulsion, sinoatrial and infrahisian conduction system disease or the association between sinus node dysfunction and atrial flutter [65]. When valvular pathology occurs, it is localized at the mitral or tricuspid valve, with regurgitation developing both pre- and postnatally and calls for urgent valve surgery [66].

There are also some other rare, but important cardiac manifestations of the presence of anti-Ro/SSA antibodies, such as sinus node dysfunction, atrial flutter, long QT interval, ventricular and junctional tachycardia and dilated cardiomyopathy [31,67]. However, the causality between these pathologies and the presence of maternal anti- Ro/SSA and anti- La/SSB antibodies is not firmly recognized.

Sinus bradycardia for example appeared in 3 of 76 fetuses (3.8%), for whom atrial rates were recorded by echocardiogram in a series of 187 fetuses with congenital heart block. Some authors consider that this disturbance is not permanent and that it could have a good prognosis [68,69]. On the other hand, persistent fetal sinus bradycardia may lead to serious cardiac complications when associated with endocardial fibroelastosis (EFE), ventricular dysfunction or AV block [69].

Dilated cardiomyopathy (DCM) occurs rarely, but has a high mortality rate [26,70]. It is characterized by an enlarged and poor-functioning left ventricle. There are two types of DCM (neonatal and late-onset). Neonatal DCM is associated with pericardial effusion, hydrops fetalis and endocardial fibroelastosis. Late-onset DCM is related to pacemaker implantation, in utero valve disease and non-European origin. [67]. Moreover, the risk factors for neonatal DCM do not predict the development of the late-onset form, which makes the long-term cardiovascular follow-up of these patients crucial for survival.

Endocardial fibroelastosis (EFE) is a condition that is characterized by diffuse thickening of the left ventricular endocardium secondary to the proliferation of fibrous and elastic tissue [71]. Nield LE et al. reported that 7 out of 13 children diagnosed with congenital heart block had EFE at presentation, while 6 developed EFE weeks to as long as five years after the diagnosis of the congenital heart block. Furthermore, 9 patients died and two underwent cardiac transplantation because of the presence of EFE [71]. The presence of EFE is associated with 51% mortality rate if it is concomitant with dilated cardiomyopathy, the mortality reaches 100% [26]. The diagnosis can be made in utero, by documenting areas of endocardial patchy echogenicity on the echocardiography examination.

#### 2.4.3. Other Transient and more Rare Manifestations

Hematologic manifestations such as anemia, neutropenia, thrombocytopenia and aplastic anemia have been described in children with congenital heart block. However, hematological involvement seems to be almost always asymptomatic [56].

Hepatosplenomegaly, asymptomatic elevated liver enzymes and cholestasis are the main hepatic manifestations observed not only in fetuses with complete heart block, but also in those with cutaneous involvement [72].

Neurologic manifestations (hydrocephalus, nonspecific white matter changes and calcification of the basal ganglia) have been also reported, but their association with anti-Ro/SSA antibodies has not yet been proven.

### 2.5. Manifestations That Suggest the Diagnosis of Neonatal Lupus

The diagnosis of neonatal lupus requires the presence of both of the following elements: (1) the mother has positive anti-Ro/SSA, anti-La/SSB or anti-RNP antibodies and (2) the fetus or newborn develops heart block/typical rash/hepatic or hematologic manifestations in the absence of another etiology [73].

For these elements to be recognized, some recommendations regarding pre- and postnatal screening of children with CHB have been formulated [74]. The prenatal evaluation focuses not only on the maternal screening for anti-Ro/SSA and anti-La/SSB antibodies, but also on in utero surveillance for heart block.

It is strongly recommended that not only symptomatic, but also asymptomatic mothers carrying positive titers of anti-Ro/La antibodies should be more intensively monitored during pregnancy with frequent echocardiographic surveillance, especially during the period from 18 to 24 weeks of gestation.

On the other hand, the American Heart Association recommends a few additional weeks of serial examinations beginning from 16 weeks until 28 weeks of gestation [75]. The most efficient modality for screening and diagnostic evaluation of this bradyarrhythmia is the *pulsed-Doppler fetal echocardiography*. This non-invasive exploration should be performed *weekly*, as it has been observed that normal sinus rhythm can progress to complete block in seven days (during the high-risk period between 18 and 24 weeks of gestation). This investigation is of great importance in measuring the mechanical PR interval from the onset of the atrial contraction to ventricular contraction. Furthermore, echocardiography is of great importance in diagnosing not only first-degree heart block, but also endocardial fibroelastosis and valvular lesions [76].

Glickstein et al. proposed a validated reproducible method to measure the fetal mechanical PR interval, the equivalent to the electric PR interval on surface electrocardiography, by echocardiography with pulsed Doppler [76,77].

**Figure 4:**
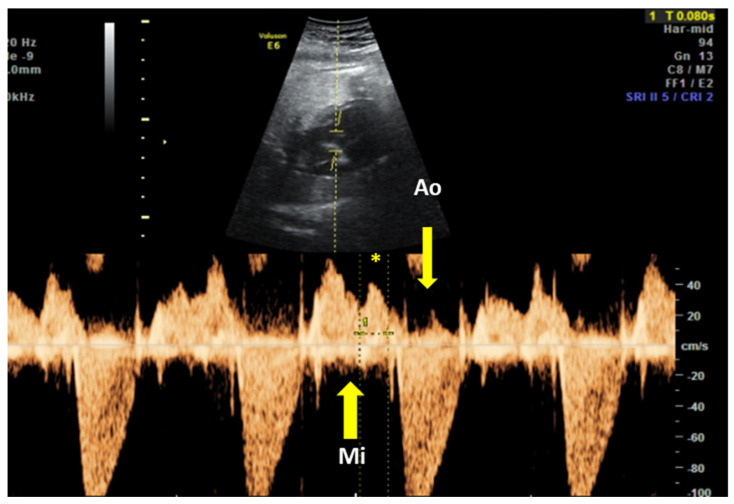
Pulsed wave Doppler trace used for mechanical PR interval measurement. Mi—transmitral flow (thick yellow arrow); Ao—transaortic flow (thin yellow arrow); (*)—normal PR interval—120 msec (between thin dotted yellow lines). Courtesy of Anca Panaitescu, Filantropia Clinical Hospital, Bucharest.

The fetal mechanical PR interval can be obtained during ultrasound examination of the fetal heart. It is recorded with the help of pulsed wave (PW) Doppler– the two-dimensional PW gate is set to 3–4 mm depending on the gestational age and placed distal to the mitral valve as such to include the origin of the left ventricle outflow tract at an angle of around 20°. With this approach, we will record spectral Doppler wave forms for both blood flows, in the mitral valve and aortic origin during a full cardiac cycle. The speed of image acquisition should be slowed (to 4–5 cm/s) in order to have a good representation of each waveform. During a normal cardiac cycle, the passive filling of the atria during generalized diastole (E wave), the active filling of ventricles during atrial systole (A wave) and blood ejection in the root of the aorta during ventricular systole will be documented (see Figure 4). The PR interval is the interval measured between the onset of atrial systole (mitral valve A-wave) and the beginning of the aortic valve flow. Normal ranges during pregnancy depend on the gestational age, fetal heart rate and sex of the baby. These correlations have been studied prospectively by A. Wojakowski et al. [78]. A mean of 122.4 ms ± SD 10.3 ms is considered normal.

In a prospective trial of dexamethasone to prevent CHB progression in women with anti-Ro by Friedman et al. the definition of “abnormal” fetal Doppler mechanical PR interval was set a priori at three SD above the normal mean, to 150 ms [79]. However, none of the conduction measurements could predict the occurrence of CHB.

*The two-dimensional ultrasound* can be used in diagnosing specific arrhythmias, in evaluating cardiac anatomy and function, as well as in searching for signs of hydrops fetalis. Arrhythmias can be further characterized by using the *M-mode ultrasonography*, which detects atrial and ventricular wall motion and the relative timing of cardiac events (see clinical case [80]. Fetal bradycardia, the result of both second- and third- heart block, can be easily detected by routine fetal auscultation.

The weekly pulsed-Doppler fetal echocardiography is the preferred method of in utero surveillance for heart block. However, if not available, there is an alternative that consists of home monitoring for fetal bradycardia, twice per day, with a handheld Doppler system [80]. The postnatal testing is recommended for mothers of neonates with heart block in the absence of causal structural abnormalities, as most cases of congenital heart blocks appear as a result of the presence of anti-Ro/SSA and/or anti-La/SSB antibodies. In addition, children up to eight months of age presenting rash and/or any degree of heart block should also be tested for the aforementioned antibodies [36,81].

## 3. Management of Positive Anti-Ro/SSA Pregnancies

### 3.1. First Pregnancy in Asymptomatic Carriers of Anti-Ro/SSA Antibodies

Specific recommendations for these categories of patients are still waiting to be formulated. There is however some evidence dating back to 2012, when Izmirly PM et al. observed that the administration of hydroxychloroquine in women with SLE can reduce the risk of development of fetal heart block. Furthermore, this therapy seems to be able to reduce the risk of developing anti-Ro/SSA related cardiac complications in patients with neonatal lupus [82,83].

As a result, many doctors are prescribing hydroxychloroquine during the first pregnancy in asymptomatic mothers carrying anti-Ro/SSA antibodies, in order to reduce the risk of developing cardiac neonatal lupus. This therapeutic attitude is also supported by the fact that in 2016, EULAR (European League Against Rheumatism) released an exhaustive analysis regarding the administration of antirheumatic agents during pregnancy and breastfeeding, including hydroxychloroquine among some other drugs which have been proved to be compatible with pregnancy [84]. Furthermore, it has been observed that the administration of this drug was not associated with an increased rate of congenital malformations [85].

### 3.2. In Utero Management of Neonatal Lupus

The treatment of first-degree heart block is the subject of many scholarly debates, as its rate of progression to more advanced block is still questioned, and a prolonged PR cannot predict the evolution toward CHB.

A Canadian prospective observational study on the effects of maternal anti-Ro/SSA and anti- La/SSB antibodies on 165 fetuses observed that the fetal AV prolongation did not predict the development of second- or third-degree heart block and questioned the strategy of early identification and treatment [86]. On the other hand, there are some small and uncontrolled case reports and series, showing that progression can occur and suggesting that treatment of first-degree heart block should be recommended.

**Figure 5:**
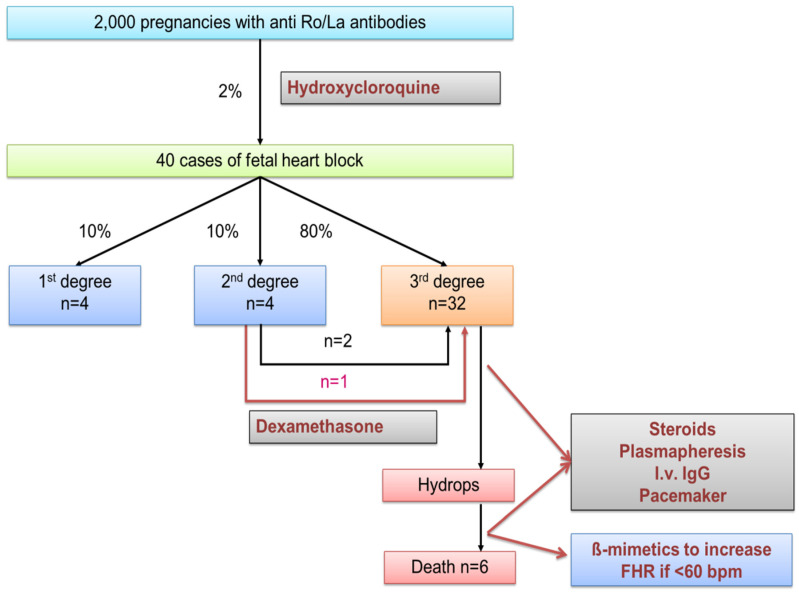
Management of pregnancies with anti-Ro/La antibodies. Adapted from Panaitescu et al. [32] FHR—fetal heart rate; I.v. IgG—intravenous immunoglobulins G.

Out of the three types of block, third-degree occurs in 80% of cases of CHB, and the rest is evenly distributed between first and second degree. The management of each type of block is different, as shown in Figure 5.

There is still debate and contradictory data regarding the specific treatment for each degree of heart block and even more regarding the prophylactic medication in the first-degree block. Although there is no current guideline, 50% of specialists would recommend starting steroids after a diagnosis of *first-degree block*. An interesting approach has been described and proposed by Buyon JP et al. [87]. The author begins by waiting for 24 h once per first-degree heart block is detected, in order to confirm that the PR interval is indeed prolonged, case in which the recommendation is maternal intake of *oral fluorinated glucocorticoids* (oral dexamethasone 4 mg per day or betamethasone 3 mg per day) for one week and fetal echocardiography monitoring. The treatment is discontinued if the first-degree block progresses to complete block and if there is no evidence of extra-nodal disease. If the block remains at first degree or reverts to normal sinus rhythm, the benefits and the risks of steroid treatment should be carefully weighed because there is still uncertainty on how long the dexamethasone should be continued. Depending on fetal echocardiography evolution, dexamethasone may be continued up to 26 weeks gestations, when the critical period reaches the end [86,88,89].

Fluorinated glucocorticoids (dexamethasone and betamethasone) are recommended in these particular situations because they are not inactivated by placental 11-beta dehydrogenase, a property which allows them to reach the fetal circulation. Nonetheless, there is limited evidence regarding their efficacy in reducing mortality in patients with cardiac neonatal lupus, while the risks of the therapy have been clearly determined not only in infants (oligohydramnios, growth restriction), but also in mothers (infections, hypertension, avascular necrosis, insulin resistance, gestational diabetes), especially with longer-term treatment [90].

*The second-degree fetal block* is known for its tendency to progress to complete heart block, although there are case series documenting that both treated and untreated patients can progress, stabilize or even revert to normal conduction [90]. A 2018 systematic review and meta-analysis of five observational studies that included 71 fetuses with second-degree immune-mediated congenital heart block concluded that the use of fluorinated glucocorticoids should not be discouraged, ”until more robust evidence is available” [90].

The presence of an *established third-degree fetal block* is an irreversible condition which implies fibrosis and calcification of the AV node. At this point, the only rational objective is the prevention of fetal hydrops and intrauterine death, as many studies have proved that the administration of fluorinated glucocorticoids does not have any effect, since the reversal of the established third-degree block has not been documented [91]. However, the transition from a normal rhythm to emergent complete CHB and from emergent CHB to established CHB can happen in < 24 h, and the treatment of the former can revert the conduction to 1:1 [92]. This reinforced the fact that in selected cases, daily HR monitoring can be crucial to diagnose and effectively treat emerging complete CHB, as established complete CHB is usually irreversible [31]. One recent study proved that home monitoring is useful in detecting the emergence of CHB [93]. Otherwise, treatment of the CHB per se was shown to be ineffective, most surviving newborn needing pacemakers [94].

Another study of 202 cases of third-degree block concluded that the therapy with the aforementioned drugs did not improve the overall survival [95]. Izmirly et al. observed after further research that there is no reason to support the use of fluorinated steroids to prevent disease progression or death in cases presenting with isolated heart block associated with anti Ro/SSA antibodies [96].

A therapy addressed to the circulating antibodies is plasmapheresis, which removes 52- and 60-kDa anti-Ro/SSA and anti-La/SSB antibodies. The second-degree block seems to recede after this treatment. Unfortunately, even if the levels of antibodies decrease, the already established third-degree block does not regress [97,98].

Using small case series, some authors described a few benefits (a small increase in the ventricular rate and improvement of cardiac function) from the administration of maternal beta-agonist therapy (salbutamol, terbutaline) when the fetal heart rate was <55 bpm [99] or <60 bpm [100].

The association between third-degree heart block and extra-nodal diseases such as endocardial fibroelastosis or cardiomyopathy was treated in several studies with fluorinated glucocorticoids and/or intravenous immunoglobulin (iv. IG), although their effectiveness is still questionable [81].

There is no conclusive evidence on the best management of pregnancies with anti-Ro and anti-La antibodies in the presence of fetal heart block. These patients are best followed by serial echocardiography and detailed anatomy ultrasound to look for further complications.

The disease is a spectrum, and some babies tolerate the CHB well up to the moment of delivery when a pace maker can be implanted, while some develop hydrops, and unfortunately die. The prognosis of hydropic fetuses with CHB is quite poor, as not even the intrauterine placement of pacemakers was not successful [101].

However, the search for novel remedies, like peptide-based therapies, is ongoing. Short non-immunogenic peptides can be used as decoy targets for pathogenic autoantibodies. They are meant to reduce antibody binding to their cellular targets (i.e., calcium channels), thus reducing the damage [39].

### 3.3. Postnatal Management of Neonatal Lupus

There are several possible clinical scenarios in the postnatal period that require a special approach. For instance, asymptomatic children born to mothers with anti-Ro/SSA and/or anti-La/SSB antibodies should be screened through electrocardiograms (ECG) and referred to a pediatric cardiologist if any abnormality is detected on the ECG tracing.

As soon as the first- or second-degree block is identified after birth, intense monitoring of the newborns is required because, as some authors observed, there is a risk of postnatal progression to third-degree block [68]. Infants born with complete heart block and with heart rates under 55 beats per minute require the implantation of a cardiac pacemaker. Unfortunately, those who are asymptomatic at birth endup also requiring a pacemaker later, during childhood or adolescence. In 2018, the ACC/AHA/HRS Guideline on the Evaluation and Management of Patients with Bradycardia and Cardiac Conduction Delay was released, including clear recommendations regarding pacemaker implantation in patients with congenital heart block [102]. The situations in which the implantation was recommended (class I) or seemed reasonable (class IIA) are described in Table 2.

In the adult population the endocardial pacemaker is the gold standard for implantation. However, in the neonatal and pediatric population the situation is different because of small caliber vessels, the risk of infection and thrombosis, the need for wire replacement, etc. Hence, the epicardial pacemaker is the preferred solution, showing excellent mid-term results in a recent study [103]. In addition, there is encouraging data to support the use of dual-chamber pacemakers instead of the single-chamber option when recommended [104].

A special mention should be reserved for newborns diagnosed with neonatal lupus and their risk of developing autoimmune and/or rheumatic disease, which is a current concern for some specialists. Fortunately, small studies proved that these complications are rare. For example, in a cohort of 49 children followed until the age of 8 years, only six patients (12%) developed an autoimmune disease (psoriasis, thyroid disease, type one diabetes mellitus, nephrotic syndrome, polyarticular juvenile idiopathic arthritis) none of which was SLE [60].

### 3.4. Prevention of Neonatal Lupus in Subsequent Pregnancies

To summarize, the risk factors for developing CHB are: positive anti-Ro/SSA and/or anti-La/SSB mothers, high titers of anti-Ro, a previous baby with CHB, cutaneous neonatal lupus in a sibling, lack of prophylactic treatment with hydroxychloroquine, predisposing fetal genes (e.g., SNPs within the HLA region [105], TGF-beta polymorphisms [25,45], IFN-stimulated genes [106]. Thus, in these situations, close monitoring and prophylactic treatment should be started as soon as possible, to prevent CHB occurrence.

Hydroxychloroquine inhibits Toll-like receptor signaling, and since there is a connection between anti-Ro, TLR 7 and fibrosis through a certain population of macrophages, it is used as prophylactic treatment [107]. Buyon et al. elaborate a recommendation regarding the treatment with hydroxychloroquine in mothers with a previously affected fetus [82]. After consulting several studies describing the efficacy of this drug in decreasing the overall risk of cardiac–neonatal lupus, the author suggests a pre-emptive treatment with hydroxychloroquine (400 mg orally once per day) in pregnant women who have previously given birth to a child with cardiac manifestations of neonatal lupus and who have anti-Ro/SSA and/or anti-LA/SSB antibodies, regardless of maternal health status (Grade 2B). This treatment should be initiated between 6 and 10 weeks gestation in women who are not already on the medication [82].

## 4. Case Report

**Figure 6:**
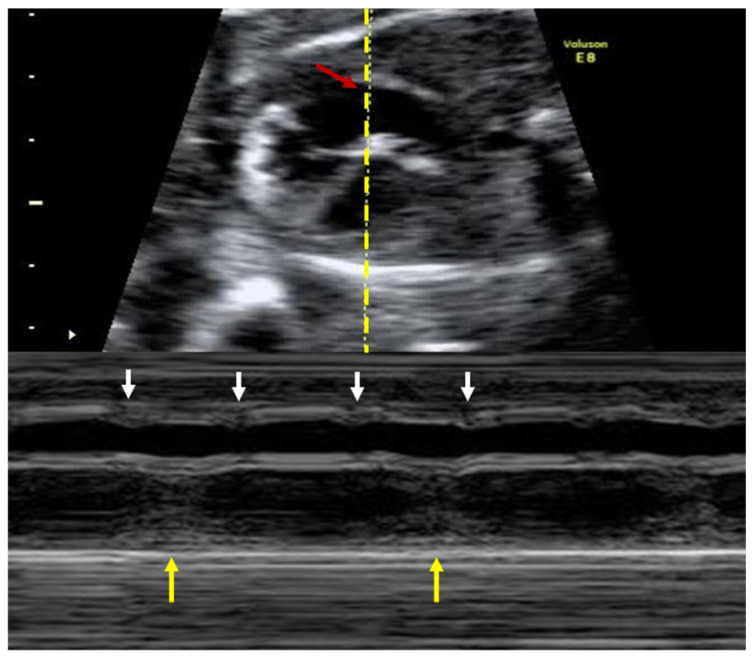
M-mode echocardiography used to demonstrate the fetal third-degree atrioventricular block. The atrial and ventricular activities are completely dissociated. Red arrow, dotted yellow line: section plane. Small white arrows: ventricular contractions. Large yellow arrows: atrial contractions. Courtesy of Anca Panaitescu, Filantropia Clinical Hospital, Bucharest.

A 28-year-old completely asymptomatic female at 23-weeks gestation was admitted for further investigations regarding a recently diagnosed third-degree AV fetal block, on a routine obstetrical visit. The total dissociation between the atrial and ventricular activity is best demonstrated by the use of M-mode echocardiography, which simultaneously records the atrial and ventricular contractions (see Figure 6).

Furthermore, fetal bradycardia, dilated and dysfunctional ventricles and even hydrops fetalis were observed by the maternal–fetal specialist during echocardiography (see Video S1).

The laboratory results indicated lymphopenia and normocytic normochromic anemia as well as hypergammaglobulinemia, positive antinuclear antibodies and high titers of anti-Ro/SSA (>200 UI/L) and anti-La/SSB antibodies (>200 UI/L). However, there was not any clinical sign of lupus. In addition, the anti-dsDNA, anti-Sm antibodies were negative, the C3 and C4 complement were normal and the urine workup was completely normal.

**Figure 7:**
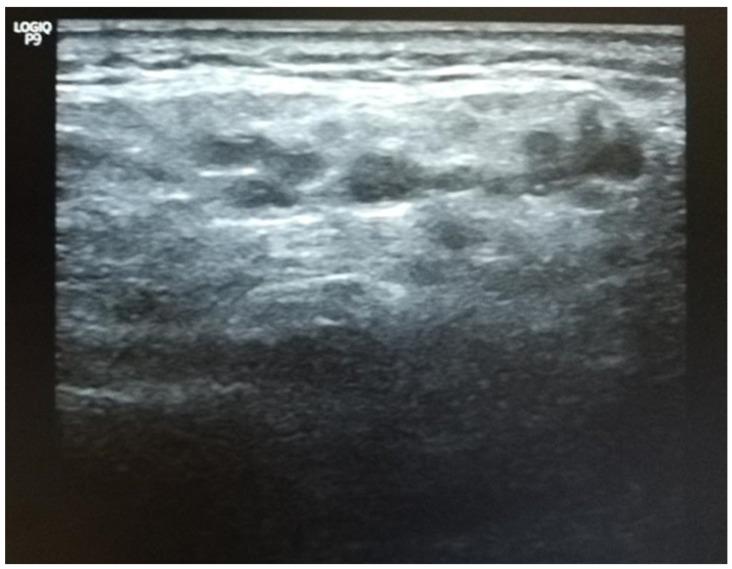
Ultrasound of the patient’s salivary glands. Grade 3, severe changes: diffuse inhomogeneity with anechoic/hypoechoic areas occupying the entire gland surface and surrounded by abnormal tissue and hyperechoic bands, corresponding to the scoring system described by S. Jousse-Joulin et al. [108]. Courtesy of Ciprian Jurcut, Internal Medicine Department, “Dr Carol Davila” Central Emergency University Military Hospital Bucharest, Romania.

Although the patient was completely asymptomatic, the Schirmer test diagnosed severe eye dryness (4 mm for the left eye and 5 mm for the right eye). In addition, the parotid glands had some echographic particularities (see Figure 7).

The diagnosis of Sjögren’s Syndrome was established based on clinical and paraclinical findings and treatment with hydroxychloroquine 200 mg per day, dexamethasone 4 mg and artificial tears was recommended to the mother.

The couple was counselled by the multidisciplinary team regarding the treatment options, poor fetal prognostic and long-term implications. Considering the high risk of fetal demise, continuation versus termination of pregnancy (TOP) was discussed. The parents opted for termination of pregnancy.

This is a typical example of an apparently asymptomatic woman, carrier of anti-Ro/La antibodies. The fetal third-degree block was the first to be identified and led to an extensive investigation and finally, to the detection of the underlying autoimmune disorder. As this first pregnancy was complicated by CHB, all subsequent pregnancies will require close monitoring starting with 16 weeks gestation. In addition, prophylactic treatment with hydroxychloroquine needs to be instituted early, as there is a high risk of CHB recurrence.

## 5. Discussion

The rheumatologists and maternal–fetal specialist concerns relate to the risk awareness, prophylaxis, early diagnosis and management of pregnancies in anti-Ro positive mothers. The pediatric cardiologist and later on the adult cardiologist, deal with the short-term and long-term consequences of the autoimmune reaction on the cardiovascular system as a whole. We conclude our review with a short discussion of the main issues as seen from these three different points of view.

### 5.1. The Rheumatologists’ Point of View

Encountering during the clinical practice young pregnant women with positive anti-Ro antibodies with such dramatic consequences, raises serious concerns and needs clear recommendations. Exhaustive, validated guidelines should include all categories of patients that need to be tested for the presence of these antibodies, especially when it comes to planning a pregnancy.

Another concern is the management of patients with positive anti-Ro antibodies, especially when it comes to the prevention of the congenital fetal heart block. More studies need to address the efficiency and safety of hydroxychloroquine administration during pregnancy and the risk-benefit ratio. Probably, in mothers at risk, hydroxychloroquine should be best initiated prior to conception. A step in the right direction is the ongoing study “Preventive Approach to Congenital Heart Block With Hydroxychloroquine (PATCH) Study” [109]. The awaited study has released up to date only a conference abstract that confirms the benefits of hydroxychloroquine prophylaxis of recurrent fetal CHB, if started as soon as the pregnancy is established. The risk reduction is significant, bringing the estimated recurrence of 18% down to 8%. Interestingly, half of the study population was asymptomatic, without SLE or SS. In addition, as per study protocol, mothers that were already taking hydroxychloroquine continued treatment [110].

Nonetheless, the awareness of the fetal complications induced by the presence of these antibodies, should lead to an increased diagnosis rate of the anti-Ro antibodies carriers, and subsequently to more intense surveillance during pregnancy.

### 5.2. The Maternal–Fetal Specialist Point of View

Women with known positive anti-Ro antibodies status planning a pregnancy should be first, referred to a specialized fetal medicine unit and a multidisciplinary approach should be initiated. Patients should be thoroughly counselled regarding the potential risks, treatment options and special monitoring required during the critical vulnerable period defined between 16 and 26 weeks. Previous obstetrical history is important, as preceding affected pregnancies increase the recurrence risk. Although best management strategy is yet to be defined, weekly fetal echocardiography and hydroxychloroquine started even before conception may reduce the risks in pregnant women with anti-Ro antibodies.

The challenge arises in asymptomatic women diagnosed with anti-Ro antibodies for the first-time during pregnancy due to fetal complications which further lead to autoimmune investigations. Congenital AV heart block occurs in 40% of the cases in fetuses with structural cardiac abnormalities such as left atrial isomerism or corrected transposition of the great arteries. In the remaining 60% of cases, an immune maternal condition is involved. Therefore, thorough fetal cardiac examination should be carried out by the maternal–fetal specialist and appropriate investigations undertaken. One should also not forget other maternal conditions such as diabetes mellitus, infection or concomitant therapies, which need to be ruled out [32].

If second or third-degree fetal heart block can be easily recognized due to fetal bradycardia, a first-degree block may be overlooked if maternal immune status is not known. A first-degree block is characterized by prolonged AV conduction with normal heart rate and translated into longer mechanical PR interval measured by simultaneous pulsed Doppler examination on mitral valve and ascending aorta. A PR interval longer than 0.15 s (3 SD above the mean) is considered a warning sign and is encountered in 30% of pregnancies with anti-Ro antibodies [111,112]. An interval between 0.13 to 0.15 s should be reviewed in 24 h for confirmation, as in most cases it is benign and spontaneously returns to normal [91], therefore its interpretation should be cautious especially when deciding steroid treatment to prevent the progression. On the other hand, there are also unpredictable cases with rapid progression from normal sinus rhythm to complete block, without being preceded by increased PR interval [91]. Despite all these limitations, most specialists recommend weekly measurements of mechanical PR interval starting from 16 weeks for patients with anti-Ro antibodies, in order to select the cases at high risk for progression to complete heart block. For diagnostic criteria and methods of diagnosis for the different types of atrioventricular block, see Table 3.

In establishing the diagnosis of CHB it is important to differentiate a second or third-degree block from blocked premature beats with bigeminy or trigemini, which is a benign condition and usually resolves spontaneously with advancing gestation. In bigeminy or trigeminy there is a shorter atrial impulse on every second, respectively third beat, while in AV block the interval between atrial impulses remains constant [117].

Other cardiac findings may be encountered and were previously reported, such as tricuspid regurgitation, sinus bradycardia, long QT interval or fetal hydrops in congestive heart failure associated with third-degree block [118]. In case of long-term steroids administration, special attention should be paid to monitor fetal growth and amniotic fluid [117].

In the absence of fetal complications, usually delivery is planned at 37 weeks gestation in specialized centers with appropriate resources for cardiac and neonatal intensive care. A new approach suggests delaying delivery until 39 weeks of gestation which may improve the neonatal outcome [106]. Pregnancy should be prolonged as much as possible to permit fetal weight gain and to reduce the peripartum complications. In case of third-degree fetal heart block and severe bradycardia, difficulties of fetal heart rate monitoring in labor and misinterpretation of signs of fetal distress inevitably impose delivery by planned cesarean section.

### 5.3. The Cardiologist Point of View

A main concern is missing some cases due to lack of heart block diagnosis, during routine check-up, in unknown anti-Ro status pregnancies. In addition, the timing of diagnosis is of particular importance, as it was shown that an earlier occurrence of CHB is associated with a higher mortality rate [31].

Another significant aspect is the association of rare, but very serious pathologies accompanying CHB, like valvular disease or DCM, which need to be swiftly diagnosed and addressed.

Last, but not least is the need for a consistent follow-up program for short and long-term consequences stemming from the passively acquired autoimmunity.

#### 5.3.1. Short-Term Management

Almost 70% of the children born with CHB need a pacemaker in the preschool years. Most of them require the intervention in the first twelve months, and the majority is concentrated within ten days after birth [31]. Hence, the indication for a pacemaker and the problematic of the type and timing of implantation is the primary matter that needs to be addressed postnatally [119]. Moreover, the pacemaker implantation leads to a set of complications of its own, like left ventricular dyssynchrony (in right ventricular leads) conducting to remodeling, dysfunction and valvular regurgitation in up to 30% of children [70,120,121,122,123].

#### 5.3.2. Long-Term Consequences and Follow-Up

There are data regarding the high risk of cardiovascular morbidity and infections in children with CHB [124]. Furthermore, there is an increased prevalence of systemic connective tissue disorder in this population, which in itself poses a threat of AV block or tachyarrhythmia later in life and requires close follow-up [124]. In this regard, a new entity was recently described, namely autoimmune channelopathies [51]. This arrhythmogenic mechanism attempts to cover the gap of unexplained sudden cardiac deaths and life-threatening arrhythmias in an otherwise structurally healthy heart. In anti-Ro positive mothers, as well as in their offspring, QTc prolongation [118,125,126,127,128] was diagnosed and linked to high titers of anti-Ro52. In addition, ventricular arrhythmias were recorded in the same group of patients [129,130,131,132].

In addition, in vitro studies suggest a connection between atrial fibrillation and anti-Ro antibodies, explained by the inhibitory effect on the α_1D_ Ca channel, which has a specific atrial distribution [133].

Regarding AV blocks and exposure to anti-Ro antibodies in utero, it appears that even children with a normal ECG at birth, which had at some point a prolonged AV interval during gestation, develop a first-degree block in early childhood [134]. It seems that the antibodies’ effects persist long after they are cleared from the bloodstream. This information supports the hypothesis that the mere presence of anti-Ro antibodies is necessary, but not enough, and additional factors play a key role. It may also explain why the electrophysiological effects do not appear in all pregnancies of anti-Ro positive mothers.

Moreover, another study describes a form of AV block with late-onset in children exposed in utero to anti-Ro antibodies [51]. In this population, it seems that there is a latent congenital damage of the conductive tissue that manifests later in life, which is antibody independent.

In addition, a retrospective study points to a possible association between the adult third-degree block of unknown etiology and anti-Ro antibodies [39,135]. Thus, there are two forms of anti-Ro associated AV block in adults: one linked to acquired autoimmunity in adult life, and another with a slow progressing congenital form in individuals exposed to anti-Ro antibodies before birth [51].

Apart from the arrhythmic consequences, it appears that adult patients and even children with lupus or juvenile-onset mixed connective tissue disease have a higher and earlier onset of diastolic dysfunction [136,137,138,139]. This is associated with the risk to develop heart failure with preserved ejection fraction over time. This pathologic entity has a similarly unfavorable prognosis as heart failure with reduced ejection fraction [140].

## 6. Conclusions

Anti-Ro antibodies, present in rheumatologic disease, but also in the clinically healthy population can trigger lifelong effects, ranging from fetal complications, like complete (irreversible block) or incomplete CHB with high in utero mortality or postnatal morbidity, to adult comorbidities.

The anti-Ro induced autoimmune reaction causes long-term fibrosis and calcification of the conductive tissue. In addition, the CHB injury mechanisms were shown to involve other factors, like fetal susceptibility that increases for every subsequent pregnancy, from a 2% incidence in the case of nulliparous mothers. The predictive value of maternal anti-Ro antibodies for CHB high-risk pregnancy is low, and other markers are lacking, making this condition difficult to efficiently monitor. Moreover, the positive anti-Ro pregnancies do not benefit from a prophylactic treatment or from an efficient therapy once CHB was diagnosed. Thus, new data from ongoing trials are highly expected, to provide both potential biomarkers and therapeutic solutions.

Creating multidisciplinary teams of trained specialists would be a highly needed initiative, as they could develop strategies for better risk awareness, administer prophylactic therapies in high-risk pregnancies, minimize undiagnosed cases and ensure appropriate postnatal treatment and follow-up.

This integrative review illustrates the current understanding of the anti-Ro antibodies associated pathologies, from the perspective of specialists involved in its management, emphasizing key issues, missing links, and possible future directions for an effective interdisciplinary approach.

## Figures and Tables

**Table 1:** Categories of patients that should be tested for anti-Ro/SSA antibodies before pregnancy.

Patients with symptoms specific to Sjögren’s syndrome: xerostomia, xerophthalmia, salivary gland enlargement [14]
Women with Sjögren’s syndrome or systemic lupus erythematosus who intend to become pregnant [13]
Patients with symptoms suggesting the diagnosis of systemic lupus erythematosus, but with a negative indirect immunofluorescence test for antinuclear antibodies [13]
Patients with rheumatoid arthritis or juvenile idiopathic arthritis [24]
Women with a history of giving birth to a child with congenital heart block or neonatal lupus [24]
Antinuclear antibody (ANA)-positive women who are planning to become pregnant

**Table 2:** Recommendations regarding pacemaker implantation in patients with congenital heart block (CHB) [102].

Congenital Heart Block AND	Class of Recommendation
Symptomatic bradycardia or low cardiac output	I
Wide QRS escape rhythm, complex ventricular ectopy or ventricular dysfunction	I
Infants with normal anatomy and a ventricular rate less than 55 beats/min	I
Infants with other structural congenital heart disease and a ventricular rate less than 70 beats/min	I
Asymptomatic adults with congenital CHB	IIa
Children beyond the first year of life with an average heart rate less than 50 beats per minute or abrupt pauses two to three times the basic R–R cycle length	IIa

**Table 3:** Heart block characteristics and diagnosis.

Block Degree		US Diagnosis	Obtained Data	FHR Limits [75,113,114,115]
1st degree		Spectral PW Doppler at the level of mitral inflow and Aortic outlet	PR > 150 ms [79]	Normal heart rate 120–160 bpm [116]
2nd degree	Type 1	M-mode; spectral PW	Progressive PR prolongation, until an atrial wave is blocked	Irregular rhythm Normal heart rate
	2:1 block		Every other atrial beat is blocked	Regular rhythm 60–80 bpm
	Type 2		Sudden block of an atrial wave	Regular rhythm Normal heart rate
3rd degree 3		M-mode	Complete A–V dissociation	<60 bpm

FHR—fetal heart rate; PW—pulsed wave; US—ultrasound.

## Supplementary Materials

The following are available online at https://www.mdpi.com/2075-4418/10/7/478/s1, Video S1: 2D- Echocardiography.
